# Transcatheter Arterial Embolization (TAE) in the Management of Bleeding in the COVID-19 Patient

**DOI:** 10.3390/medicina59061062

**Published:** 2023-06-01

**Authors:** Roberto Minici, Federico Fontana, Massimo Venturini, Giuseppe Guzzardi, Agostino Siciliano, Filippo Piacentino, Raffaele Serra, Andrea Coppola, Pasquale Guerriero, Biagio Apollonio, Rita Santoro, MGJR Research Team, Luca Brunese, Domenico Laganà

**Affiliations:** 1 Radiology Unit, Dulbecco University Hospital, 88100 Catanzaro, Italy; miniciroberto@gmail.com (R.M.); agostino.siciliano@tiscali.it (A.S.); 2 Diagnostic and Interventional Radiology Unit, ASST Settelaghi, Insubria University, 21100 Varese, Italy; massimo.venturini@uninsubria.it (M.V.); filippo.piacentino@asst-settelaghi.it (F.P.); andrea.coppola@asst-settelaghi.it (A.C.); 3 School of Medicine and Surgery, Insubria University, 21100 Varese, Italy; 4 Radiology Unit, Maggiore della Carità University Hospital, 28100 Novara, Italy; giuguzzardi@gmail.com; 5 Vascular Surgery Unit, Department of Medical and Surgical Sciences, Magna Graecia University of Catanzaro, Dulbecco University Hospital, 88100 Catanzaro, Italy; rserra@unicz.it; 6 Radiology Unit, Santobono-Pausilipon Hospital, 80129 Naples, Italy; pasqualeguerriero@gmail.com; 7 Department of Medicine and Health Sciences, University of Molise, 86100 Campobasso, Italy; luca.brunese@unimol.it; 8 Radiology Unit, San Timoteo Hospital, 86039 Termoli, Italy; bapollonio@sirm.org; 9 Haemophilia and Thrombosis Center, Dulbecco University Hospital, 88100 Catanzaro, Italy; ritacarlottasantoro@gmail.com; 10 Magna Graecia Junior Radiologists Research Team, 88100 Catanzaro, Italy; radiologyumg@gmail.com; 11 Scientific Committee of the Italian National Institute of Health (Istituto Superiore di Sanità, ISS), 00161 Rome, Italy; 12 Radiology Unit, Department of Experimental and Clinical Medicine, Magna Graecia University of Catanzaro, Dulbecco University Hospital, 88100 Catanzaro, Italy

**Keywords:** COVID-19, bleeding, TAE, embolization, hemorrhage, coagulopathy, embolic agents, endovascular

## Abstract

*Background and Objectives*: Increasing attention is being paid to the coagulation disorders associated with SARS-CoV-2 infection. Bleeding accounts for 3–6% of COVID-19 patient deaths, and is often a forgotten part of the disease. The bleeding risk is enhanced by several factors, including spontaneous heparin-induced thrombocytopenia, thrombocytopenia, the hyperfibrinolytic state, the consumption of coagulation factors, and thromboprophylaxis with anticoagulants. This study aims to assess the efficacy and safety of TAE in the management of bleeding in COVID-19 patients. *Materials and Methods*: This multicenter retrospective study analyzes data from COVID-19 patients subjected to transcatheter arterial embolization for the management of bleeding from February 2020 to January 2023. *Results*: Transcatheter arterial embolization was performed in 73 COVID-19 patients for acute non-neurovascular bleeding during the study interval (February 2020–January 2023). Coagulopathy was observed in forty-four (60.3%) patients. The primary cause of bleeding was spontaneous soft tissue hematoma (63%). A 100% technical success rate was recorded; six cases of rebleeding resulted in a 91.8% clinical success rate. No cases of non-target embolization were observed. Complications were recorded in 13 (17.8%) patients. The efficacy and safety endpoints did not differ significantly between the coagulopathy and non-coagulopathy groups. *Conclusions*: Transcatheter Arterial Embolization (TAE) is an effective, safe and potentially life-saving option for the management of acute non-neurovascular bleeding in COVID-19 patients. This approach is effective and safe even in the subgroup of COVID-19 patients with coagulopathy.

## 1. Introduction

The World Health Organization (WHO) declared Coronavirus disease 2019 (COVID-19) a pandemic due to the significant impact on the health system inflicted by the serious acute respiratory syndrome of Coronavirus 2 (SARS-CoV-2) [1,2,3]. Although the main cause of death of the COVID-19 patient is represented by pneumonia and its related complications [4,5,6], increasing attention is paid to the coagulation disorders associated with SARS-CoV-2 infection [7,8,9,10]. Endothelial dysfunction plays a pivotal role in leading the COVID-19 patient to a hypercoagulable state, with up to 95% of COVID-19 patients presenting with an elevated D-dimer, a prolonged prothrombin time, a low platelet count, and other laboratory abnormalities [11,12].

However, bleeding is often the forgotten side of the disease, and the bleeding risk is enhanced by several factors, including spontaneous heparin-induced thrombocytopenia (HIT) due to the endogenous release of heparin, thrombocytopenia, a hyperfibrinolytic state, the consumption of coagulation factors, thromboprophylaxis with anticoagulants, disseminated intravascular coagulation (DIC), and sepsis-induced coagulopathy (SIC) [7,9,13,14,15,16,17,18]. Bleeding accounts for 3–6% of COVID-19 patient deaths [19,20].

Transcatheter arterial embolization (TAE) is now a milestone in the treatment of clinically relevant major acute bleeding [21,22,23]. In patients with coagulation disorders undergoing TAE, significant heterogeneity of data regarding technical success, risk of rebleeding and choice of embolic agent, persists [24,25,26,27]. Considering the high incidence of coagulation disorders in COVID-19, it is worthy of note that to date few studies have selectively investigated TAE in COVID-19 patients, with a preponderance of case reports and short case series [28,29,30,31,32,33,34].

This multicenter retrospective cohort study aims to assess the efficacy and safety of TAE in the management of bleeding in the COVID-19 patient.

## 2. Materials and Methods

### 2.1. Study Design

**Figure 1:**
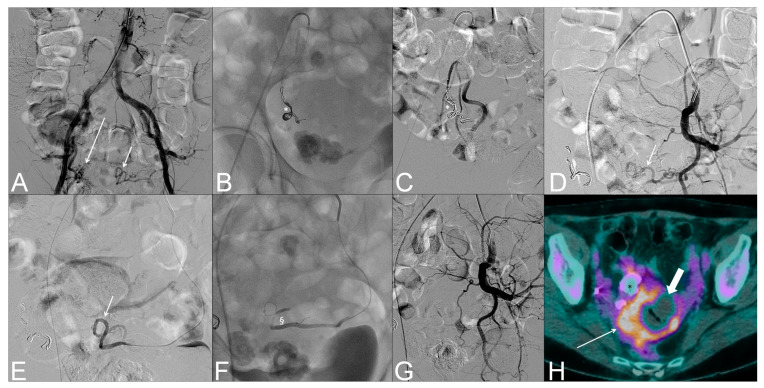
Digital Subtraction Angiography (**A**–**G**) and subsequent 18-FDG-PET/CT (**H**) of a 70-year-old woman with recently diagnosed endometrial cancer, COVID-19 infected, presenting at our institution’s Emergency Department with severe metrorrhagia and anemization. (**A**) Aortography showing pathological uterine angiogenesis (long arrow) fed by the right uterine artery and the left hypertrophic uterine artery (short arrow). (**B**) Coils (*), gelfoam, and microsphere embolization of the right uterine artery. (**C**) Right uterine angiography demonstrating exclusion of the pathological uterine angiogenesis; radiopaque coils (*) can be seen. (**D**,**E**) Left hypogastric artery (**D**) and super-selective uterine artery (**E**) angiographies demonstrating the left hypertrophic uterine artery after coils embolization of the right uterine artery. (**F**) SquidPeri 34 (§) embolization of the left uterine artery. (**G**) Left uterine angiography demonstrating exclusion of the pathological uterine angiogenesis H. Post embolization 18-FDG-PET/CT showing inhomogeneous increased uptake (thin arrow) at the level of the known uterine malignancy with a central non-hypermetabolic region with air bubbles within (thick arrow), consistent with central necrosis and residual disease.

**Figure 2:**
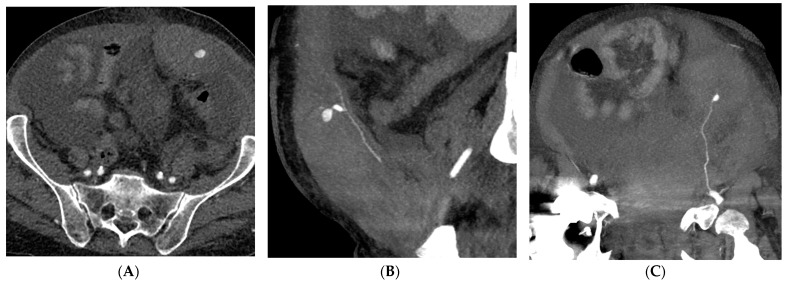

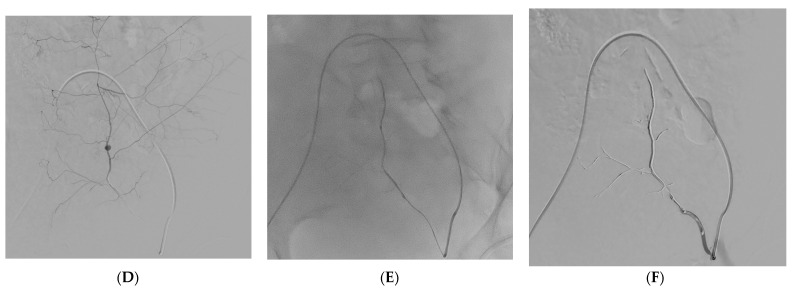
A 57-year-old man with coagulopathy, COVID-19, abdominal pain, and acute anaemia. (**A**–**C**) CT angiography demonstrating a rectus sheath hematoma with a pseudoaneurysm of the left inferior epigastric artery depicted in the oblique sagittal and coronal planes by maximum intensity projection reformatting. (**D**) Digital Subtraction Angiography showing selective catheterization and pseudoaneurysm of the inferior epigastric artery. (**E**,**F**) Fluoroscopy showing the Onyx 34 cast distributed along the inferior epigastric artery and Digital Subtraction Angiography confirming successful embolization.

This multicenter study (Pugliese-Ciaccio Hospital, Catanzaro, Italy; Circolo Hospital, Varese, Italy; Maggiore della Carità University Hospital, Novara, Italy; Mater Domini University Hospital, Catanzaro, Italy; Cardarelli Hospital, Campobasso, Italy; San Timoteo Hospital, Termoli, Italy) consisted of a retrospective analysis of data from consecutive COVID-19 patients who underwent transcatheter arterial embolization for the management of bleeding from February 2020 to January 2023 (Figure 1 and Figure 2). The inclusion criteria were (I) acute non-neurovascular bleeding requiring angioembolization in accordance with the SIR guidelines for TAE and in accordance with a laboratory, clinical and imaging evaluation performed by the interventionists [23]; (II) a positive test for severe acute respiratory syndrome coronavirus 2 infection through a reverse-transcriptase polymerase chain reaction assay on a nasopharyngeal swab; (III) be of legal age (≥18 years); and (IV) assessment by a multidisciplinary team of anesthesiologists, interventional radiologists, and surgeons. The exclusion criteria were: (I) women who were expecting or nursing; (II) a platelet count of 20,000 or less platelets per μL and an unwillingness to have a transfusion, based on SIR recommendations for low bleeding risk interventions requiring arterial access [35]; (III) with regard to femoral access, an international normalized ratio (INR) greater than or equal to 1.8, and for radial access, an international normalized ratio (INR) greater than or equal to 2.2, based on SIR recommendations for low bleeding risk interventions requiring arterial access [35]; (IV) known hypersensitivity to embolic agents; and (V) bleeding in the internal carotid artery or one of its branches.

Because the study was retrospective in nature, ethics committee approval was not necessary. The Helsinki Declaration was followed in conducting the study. Prior to having endovascular therapy, each patient provided written informed consent.

### 2.2. Treatment

A CT-angiography (CTA) scan was performed before treatment, except in cases selected on the basis of international recommendations or expert opinions (e.g., according to WSES recommendations, patients with pelvic injuries who are hemodynamically unstable [36]; upper gastrointestinal bleeding refractory to endoscopic treatment [37]). An expert interventional radiologist (at least 5 years of experience) carried out the endovascular intervention in specialized catheterization labs. A high-level infection protection protocol was adopted before, during, and after the endovascular procedure, similar to what was suggested by Ierardi et al. [38]. Prior to the super-selective catheterization of the bleeding/pseudoaneurysm-feeding arteries, a diagnostic angiography was always performed. The choice of embolic agent depended on operator preference. The embolic agent was prepared and injected under fluoroscopic guidance according to the instructions for use. The microcatheter was never reused or washed after the use of a non-adhesive liquid embolic agent (NALEA) or N-butyl cyanoacrylate (NBCA). In the absence of angiographic signs of bleeding, a blind embolization was performed, typically guided by CTA or endoscopic findings. Postembolization angiography was used to evaluate the technical success and non-target embolization, taking into consideration any potential collateral circulation depending on the anatomical site of the bleeding. When necessary, the anesthesiologist sedated the patient during the intervention to increase comfort, and an analgesic treatment was given afterwards. Before being discharged from the hospital and one month following TAE, all patients had a clinical evaluation and follow-up imaging.

### 2.3. Outcomes and Definitions

The rate of technical success served as the primary efficacy endpoint. It was determined that the clinical success rate would be used as a secondary efficacy endpoint. The rate of complications served as the primary safety endpoint. As secondary safety endpoints, the rate of non-target embolization and the rate of serious consequences, evaluated in accordance with the 2003 SIR classification [39], were chosen.

The Society for Interventional Radiology’s reporting guidelines for percutaneous transcatheter embolization were followed unless otherwise stated [39]. The International Society on Thrombosis and Haemostasis interim guidance on the recognition and management of coagulopathy in COVID-19 was used to define the coagulopathy subgroup [40]: prothrombin time (PT) prolongation above laboratory reference values (may vary depending on the reagent used), D-dimer ≥ 1.5 mg/L, fibrinogen < 2 g/L or less than 100,000 platelets per μL. Taking into account the timeframes noted in the CT report and the surgical operating log, the CT-to-groin time, procedure time, and CT-to-embolization time were determined. The 2017 SIR classification [41], the 2003 SIR classification [42], and the CIRSE classification [43] were used to grade TAE-related complications.

### 2.4. Statistical Analysis

All randomized patients who underwent at least one embolization treatment were included in the analysis as the Modified Intention-To-Treat population [44,45]. The data’s assumed normality was checked using the Kolmogorov–Smirnov and Shapiro–Wilk tests. Frequency (% value) is the presentation format for categorical data [46]. Data with a continuous normal distribution are shown as mean ± standard deviation. Data that are continuous but not normally distributed are shown as the median (first to third quartile) [47,48]. Continuous normally distributed data were evaluated using an unpaired Student’s *t*-test, whilst categorical and continuous not normally distributed data were evaluated using the Chi-squared/Fisher’s exact tests and the Mann–Whitney test, respectively. [49,50,51]. The aforementioned tests were deemed statistically significant at a *p*-value of 0.05. The data were maintained in an Excel spreadsheet (Microsoft Inc, Redmond, Wash) and the statistical analyses were performed on an intention-to-treat basis, using SPSS software (SPSS, v. 22 for Windows; SPSS Inc., Chicago IL, USA) and R/R Studio software.

## 3. Results

During the study interval (February 2020–January 2023), 73 COVID-19 patients underwent transcatheter arterial embolization for acute non-neurovascular bleeding. Coagulopathy was observed in forty-four (60.3%) patients. CT-angiography was carried out in 69 (94.5%) cases; in each of these scans, bleeding was found. On CT scans, the average hematoma volume was 356.2 (±309.4) mL. Forty-seven (64.4%) patients were on anticoagulant therapy. The preprocedural laboratory values presented are the last values recorded just prior to the endovascular procedure. The details are given in Table 1.

A total of 73 transcatheter arterial embolizations were done. One instance required a blind embolization (i.e., the embolization of a gastroduodenal artery guided by the clip released by the endoscopist), since there was no bleeding visible on x-ray angiography (XA) in that case. The abdomen (67.1% of all bleeding sites) and pelvis (12.3%) were the most frequent hemorrhage locations. Spontaneous soft tissue hematoma was the main (63%) source of bleeding. The most commonly used embolic agent was PVA particles (56.2%). In 16 (21.9%) cases, a combination of coils and a gelatin sponge was used. During embolizations, the average amount of iodinated contrast medium utilized was 35.3 (9.5) mL. The ratio of the average contrast volume to creatinine clearance was 0.7 (0.6). The common femoral artery (74%) was the most often used vascular access site. The total dose area product and cumulative air kerma measurements of the radiation exposure were 162 (60.8) mGy and 25.4 (9.5) Gy/cm^2^, respectively.

The procedure data are detailed in Table 2.

A 100% technical success rate was recorded; six cases of rebleeding resulted in a 91.8% clinical success rate. No cases of non-target embolization were noted. Complications were recorded in 13 (17.8%) patients. With just four episodes of hematoma, the risk of vascular access site complications (VASCs) was 5.5%. A total of 10 (13.7%) patients had procedure-related grade 2 events (four access site hematomas, one post-embolization syndrome, and five abscesses); one (1.4%) patient had procedure-related grade 3 events (acute pancreatitis), and two (2.7%) patients had procedure-related grade 5 events, according to the 2017 SIR classification for complications [42]. The 30-day bleeding-related mortality was 2.7%, which was the result of one case of disseminated intravascular coagulation (DIC) and one case of hypovolemic shock and Multiple Organ Dysfunction Syndrome (MODS).

The details are provided in Table 3.

BMI, platelet count, procedure time, fluoroscopy time, technical success, clinical success, rebleeding complications, and 30-day bleeding-related mortality did not differ significantly between the coagulopathy and non-coagulopathy groups. INR, D-Dimer, anticoagulant therapy, hematoma volume, cause of bleeding, and CT-to-groin time differed significantly between the coagulopathy and non-coagulopathy groups.

A comparison of data between patients with and without coagulopathy is reported in Table 4.

## 4. Discussion

In this multicenter retrospective cohort study, TAE was effective in the management of acute non-neurovascular bleeding in COVID-19 patients.

A 100% technical success rate was recorded; six cases of rebleeding resulted in a 91.8% clinical success rate, and they were all successfully retreated with TAE. These results are in keeping with other reports evaluating TAE in COVID-19 patients with acute bleeding [28,29,30,33,52].

Ierardi et al. reported a clinical success rate of 90.9% in their case series of 11 consecutive COVID-19 patients that underwent TAE for gastrointestinal bleeding. Hypertension, cancer, and preadmission anticoagulation therapy were found to be the most frequent conditions associated with gastrointestinal bleeding [29]. According to Abate et al. and Dohan et al., the incidence of spontaneous muscle hematoma is about four times higher in patients with COVID-19 compared to non-COVID patients on anticoagulant therapy [52,53]. In COVID-19 patients with spontaneous muscle hematoma, a mortality rate of 32.4% was reported [52], indicating that the occurrence of spontaneous soft tissue bleeds is a crucial event that may dramatically worsen the prognosis of COVID-19 patients. Lalatovic et al. reported four cases of retroperitoneal hemorrhages in COVID-19 patients treated by TAE, with one death caused by bleeding-related cardiovascular failure [30]. In a recent case series by Lopez-Martinez et al., 10 spontaneous soft tissue hematomas in COVID-19 patients were all successfully managed with TAE, and no cases of rebleeding were observed [28]. Tiralongo et al. reported 10 cases of spontaneous retroperitoneal hematoma in COVID-19 patients treated by TAE with coils or a gelatin sponge or a combination of both, with a clinical success rate of 70% [33]. Lucatelli et al. highlighted that TAE is superior to conservative management for the treatment of soft tissue hematomas in COVID-19 patients with decreased hemoglobin; moreover, the delay in performing TAE can worsen the prognosis, and often the interruption of anticoagulant therapy is not a viable option in severely ill COVID-19 patients [31,32]. In our study, a high percentage of bleeds were classified as spontaneous soft tissue hematomas, thus making our results consistent with other studies in the field. Hence, we can speculate that, despite the lack of specific guidelines, TAE represents an effective treatment option for spontaneous soft tissue hematomas, even in the COVID-19 patient. Finally, our results are in keeping with those reported in other investigations for acute non-neurovascular bleeding in non-COVID patients [39,54,55,56,57].

Several investigations have explored the role of angiography in detecting and treating spontaneous anticoagulant-related bleeding [53,58,59,60,61,62]. Dohan et al. reported a clinical success rate of 83% in their series of 36 consecutive patients undergoing anticoagulation-related soft-tissue bleeding embolization [53]. Our results are consistent with these findings, and demonstrate that TAE is highly effective for the treatment of acute non-neurovascular bleeds in COVID-19 patients, both with and without coagulopathy. Furthermore, four cases of early rebleeding (66.7%) occurred in patients with coagulopathy undergoing TAE with only a gelatin sponge or coils. Loffroy et al. demonstrated that the use of coils as the sole embolic agent and the presence of coagulopathy are independent predictors of early rebleeding [26]. Previous reports support the hypothesis that embolization with a gelatin sponge or coils only is less effective in patients with coagulopathy, and liquid embolics do not suffer from this limitation [24,25,26,27,63,64]. Lopez-Martinez et al. successfully embolized seven cases of spontaneous soft tissue hematomas in anticoagulated COVID-19 patients using Onyx-18 (Covidien Medtronic, Irvine, CA, USA), an ethylene-vinyl alcohol (EVOH)-based non-adhesive liquid embolic agent (NALEA), without the occurrence of rebleeding [28]. Lucatelli et al. stressed that using an adjunctive embolic agent (gelatin sponge or glue) was helpful in enhancing clot formation in COVID-19 bleeding [32]. Interestingly, our findings fuel speculation about the selection of the best embolic agent in COVID-19 patients with coagulopathy undergoing TAE. Further studies in patients with coagulopathy are required to comprehensively answer this research question, although we believe it is acceptable and prudent to avoid using coils or gelatin sponges alone, if possible, in COVID-19 patients with coagulopathy.

Computed tomography angiography (CTA) was able to identify the vascular source of bleeding in 95.2% of cases of spontaneous soft tissue hematoma in a recent study by Dohan et al. [65]. Non-enhanced computed tomography (NECT) shows the size and location of the hematoma [28,66]. The hematoma volume correlates with the outcome, and is a key factor in the decision-making process for referral to angiography [67,68]. Several reports have shown that the presence of active bleeding on the CTA-arterial phase correlates with the severity of spontaneous muscle hematomas and with the failure of conservative management [53,60,62,69,70]. Tiralongo et al. highlighted that CTA has revealed signs of active bleeding in 90% of cases of spontaneous retroperitoneal hematoma in COVID-19 patients [33]. If neither CTA nor DSA shows signs of bleeding, a blind embolization can be performed, as it guarantees efficacy and safety comparable to targeted embolization in spontaneous abdominal wall hematomas [71]. In our investigation, CTA seemed very useful for TAE planning, and all patients underwent CTA before TAE, except in selected cases. According to WSES guidelines, patients with hemodynamically unstable pelvic fractures can be referred directly for angiography to possibly perform TAE [36]. Loffroy et al. recently reiterated that CTA is not necessarily needed in recurrent upper gastrointestinal bleeding after endoscopic treatment, as the accurate location of the bleeding site is already known, and the time to angiography is a prognostic factor for clinical failure [37]. Hence, the preliminary execution of the CTA represents a key point for the management of acute bleeding except in selected cases in which the patient can be directly referred for angiography.

In our study, TAE has shown a good safety profile in the treatment of acute non-neurovascular bleeding in COVID-19 patients. The thirty-day bleeding-related mortality was 2.7%, linked to one case of DIC and one case of hypovolemic shock and MODS. Furthermore, other similar studies already published in the field have shown similar safety data, but with a much smaller sample size, meaning that some rarer complications may not have occurred [28,29,30,32,33], thus making our results even more interesting. In a recent case series on the peripheral embolization of acute gastrointestinal hemorrhages in COVID-19 patients, Ierardi et al. observed a 0% mortality rate at 30 days, a 9% rebleeding rate, and an 18.2% minor complication rate, including a groin hematoma and an ischemic rectal ulcer that were both managed conservatively [29]. According to Cavaliere et al., although an endoscopy could be curative for the management of upper gastrointestinal bleeding, the risks may outweigh the benefits in severely ill patients with COVID-19 pneumonia [72], thus giving TAE a pivotal role when conservative treatment fails [29]. Lalatovic et al. reported a death from bleeding-related cardiovascular failure in a series of four COVID-19 patients undergoing TAE with microspheres (Hydropearl™, Terumo) for retroperitoneal hemorrhage [30]. No episodes of rebleeding and an infected hematoma requiring percutaneous drainage were observed by Lopez-Martinez et al. in 10 cases of TAE for soft tissue hematomas in COVID-19 patients [28]. Lucatelli et al. reported two deaths (14.3%) from bleeding-related cardiovascular failure in a cohort of 14 COVID-19 patients undergoing TAE for spontaneous soft tissue hematoma [32]. Tiralongo et al. observed 4% minor complications (dissection flap of the deep circumflex iliac artery) in their series on the embolization of spontaneous retroperitoneal hematoma including COVID-19 patients [33]. The safety outcomes, including VASCs, are in keeping with other studies in the field of endovascular treatments and TAEs [54,73,74,75,76,77,78,79,80,81,82,83]. Furthermore, our results exceed the Society of Interventional Radiology’s reported rates and suggested thresholds of adverse events for TAE [39]. Finally, this multicenter retrospective cohort investigation confirms that TAE is a safe treatment option for managing acute non-neurovascular bleeding in patients with COVID-19, including the subgroup of patients with coagulopathy, and it does not show a different safety profile compared to TAE performed in non-COVID patients.

The absence of a control group, the retrospective nature of the research, the diversity of indications, the short-term follow-up, and the dearth of data in the literature that would have allowed us to assess the consistency and congruence of the data given are all limitations of the study.

## 5. Conclusions

To the best of our knowledge, this is one of the largest multicenter cohort studies to date that investigated the efficacy and safety of Transcatheter Arterial Embolization (TAE) in COVID-19 patients.

Hence, the results of the current investigation demonstrate that Transcatheter Arterial Embolization (TAE) is an effective, safe and potentially life-saving option for the management of acute non-neurovascular bleeding in COVID-19 patients. This approach is effective and safe, even in the subgroup of COVID-19 patients with coagulopathy.

Further studies are warranted to better understand the impact on outcomes of some factors, such as the choice of the best embolic agent, the interruption of anticoagulant therapy. and the time to angiography in COVID-19 patients with coagulopathy.

## Figures and Tables

**Table 1:** Population data.

Variables	All Patients (*n* = 73)
Age (years)	65.8 (±15.4)
Sex (M/F)	48 (65.8%)/25 (34.2%)
BMI	26 (±3.6)
eGFR (mL/min)	66.4 (±25)
CKD Stage	2 (1–3)
INR	1.3 (±0.3)
aPTT (s)	37.5 (±4.9)
PT (s)	13.9 (±2.6)
D-Dimer (mg/L)	1.2 (±0.7)
Fibrinogen (g/L)	1.9 (±0.6)
Platelet count (No. ×10^3^/μL)	367.4 (±99)
Coagulopathy (no/yes)	29 (39.7%)/44 (60.3%)
Hemoglobin (g/dL)	7.7 (±0.8)
CT-angiography execution	69 (94.5%)
Bleeding on CT-angiography	69 (94.5%)
Hematoma volume (mL)	356.2 (±309.4)
Antiplatelet therapy	12 (16.4%)
- Single	9 (12.3%)
- Dual	3 (4.1%)
Anticoagulant therapy	47 (64.4%)
Antiplatelet AND Anticoagulant therapy	1 (1.4%)
Antiplatelet OR Anticoagulant therapy	57 (78.1%)

**Table 2:** Procedure data.

Variables	All Patients (*n* = 73)
Bleeding on XA	72 (98.6%)
Blind embolization	1 (1.4%)
Site of bleeding	
- Pelvic	9 (12.3%)
- Upper GI	1 (1.4%)
- Lower GI	2 (2.8%)
- Abdomen	49 (67.1%)
- Thorax	8 (11%)
- Neck	2 (2.7%)
- Limbs	2 (2.7%)
Number of embolized vessels	1.2 (±0.5)
Cause of the bleeding	
- Trauma	18 (24.7%)
- Spontaneous soft tissue hematoma	46 (63.0%)
- Others (tumors, diverticula, ulcers, etc.)	9 (12.3%)
Embolic agent	
- PVA	41 (56.2%)
- Coils + Gelatin sponge	16 (21.9%)
- Onyx or Squid	12 (16.4%)
- Coils	2 (2.75%)
- Gelatin sponge	2 (2.75%)
Intraoperative unfractionated heparin (IU)	
- No	67 (91.8%)
- 2000 IU	4 (5.5%)
- 3000 IU	2 (2.7%)
Intraoperative contrast medium (mL)	35.3 (±9.5)
Volume of contrast to creatinine clearance ratio	0.7 (±0.6)
Vascular access site	
- Femoral	54 (74%)
- Radial	16 (21.9%)
- Brachial	3 (4.1%)
Sheath diameter, 4F/5F/6F/≥7F	9 (12.3%)/59 (80.8%)/5 (6.8%)/0 (0%)
Angiography injection technique (manual/powered)	40 (54.8%)/33 (45.2%)
CT-to-groin time (min)	41.8 (±48.9)
Procedure time (min)	28.3 (±8.4)
CT-to-embolization time (min)	70.1 (±48.8)
Fluoroscopy time (min)	7.7 (±3)
Cumulative air kerma (mGy)	162 (±60.8)
Dose area product (DAP) (Gy/cm2)	25.4 (±9.5)

**Table 3:** Outcome data.

Variables	All Patients (*n* = 73)
Technical success	73 (100%)
Clinical success	67 (91.8%)
Vascular access site hemostasis	
- Manual compression	68 (93.2%)
- Vascular closure device	5 (6.8%)
Units of packed red blood cells transfused per patient	1 (±0.6)
Rebleeding	6 (8.2%)
Non-target embolization	0 (0%)
Complications	13 (17.8%)
Vascular access-site complications (VASCs)	4 (5.5%)
Complications, according to SIR classifications	
- None	60 (82.2%)
- Minor (grade 1–2)	10 (13.7%)
- Major (grade 3–4–5)	3 (4.1%)
Complications, according to CIRSE classification	
- Grade 0	60 (82.2%)
- Grade 3	11 (15.1%)
- Grade 6	2 (2.7%)
Treatment required for complications	
- None	60 (82.2%)
- Medical	8 (11.0%)
- Interventional	5 (6.8%)
- Surgical	0 (0%)
30-day bleeding-related mortality	2 (2.7%)

**Table 4:** Comparison of data between patients with and without coagulopathy.

Variables	Group 1 (*n* = 44) Patients with Coagulopathy	Group 2 (*n* = 29) Patients without Coagulopathy	*p* Value
BMI	25.8 (±3.2)	26.1 (±3.9)	0.5856
INR	1.4 (±0.3)	1.1 (±0.1)	<0.0001
D-Dimer (mg/L)	1.7 (±0.2)	0.4 (±0.2)	<0.0001
Platelet count (No. ×10^3^/μL)	344.7 (±119.9)	401.8 (±33.2)	0.0543
Anticoagulant therapy	43 (97.7%)	4 (13.8%)	<0.0001
Hematoma volume (mL)	417.5 (±285.1)	206.2 (NA)	0.0001
Cause of the bleeding			<0.0001
- Spontaneous soft tissue hematoma	44 (100%)	2 (6.9%)	
- Others	0 (0%)	27 (93.1%)	
CT-to-groin time (min)	51.1 (±58.5)	25.4 (NA)	0.0316
Procedure time (min)	28.2 (±9.3)	28.4 (±7)	0.7432
Fluoroscopy time (min)	7.5 (±3)	7.9 (±2.9)	0.7236
Technical success	44 (100%)	29 (100%)	1
Clinical success	41 (93.2%)	26 (97.8%)	0.6762
Rebleeding	41 (93.2%)	26 (89.6%)	0.6762
Complications	6 (13.6%)	7 (24.1%)	0.3499
30-day bleeding-related mortality	1 (2.3%)	1 (3.4%)	1

## Data Availability

The data presented in this study are available on request from the corresponding author. The data are not publicly available due to privacy issues.

## Abbreviations

APTT: activated partial thromboplastin time; CKD: chronic kidney disease; COVID-19: coronavirus disease 2019; CTA: CT-angiography; DAP: dose-area product; DIC: disseminated intravascular coagulation; eGFR: estimated Glomerular Filtration Rate; EVOH: ethylene-vinyl alcohol; HIT: heparin-induced thrombocytopenia; INR: international normalized ratio; MODS: Multiple Organ Dysfunction Syndrome; NALEA: non-adhesive liquid embolic agent; NBCA: N-butyl cyanoacrylate; NECT: non-enhanced computed tomography; PT: prothrombin time; PVA: polyvinyl alcohol; SARS-CoV-2: serious acute respiratory syndrome of Coronavirus 2; SIC: sepsis-induced coagulopathy; TAE: transcatheter arterial embolization; VASCs: vascular access site complications; XA: X-ray angiography.
